# 62. Secondary Prophylaxis in *Clostridioides difficile* Infections: a Closer Look at Outcomes

**DOI:** 10.1093/ofid/ofab466.264

**Published:** 2021-12-04

**Authors:** Rubi Rodriguez, Surafel Mulugeta, Darius Faison, Rachel Kenney, Susan L Davis, Susan L Davis

**Affiliations:** 1 Henry Ford Wyandotte Hospital, Detroit, Michigan; 2 Henry Ford Hospital, Detroit, MI; 3 Henry Ford Wyandotte, Wyandotte, Michigan; 4 Wayne State University, Detroit, MI

## Abstract

**Background:**

*Clostridioides difficile* infection (CDI) is an urgent public threat and carries a 25% chance of recurrence (rCDI). Data on safety and efficacy of oral vancomycin prophylaxis (OVP) in reducing rCDI is limited. We implemented a best practice advisory (BPA) to alert the prescriber and antibiotic stewardship (ASP) team for patients with CDI in the previous 60 days being initiated on systemic antimicrobials. The alert states “Don’t use antibiotics in patients with recent CDI without convincing evidence of need. Antibiotics pose a high risk of recurrence”. ASP team would recommended OVP if antibiotics are continued. This study evaluated the impact of the BPA alert on OVP prescribing and patient outcomes.

**Methods:**

IRB approved, retrospective, observational cohort study comparing patients who received OVP to no OVP. Inclusion: adults with history of laboratory confirmed CDI, ≥ 14 days post-CDI treatment completion, BPA from 3/7/19 – 3/31/20, receiving non-CDI systemic antimicrobials, and without history of bezlotoxumab infusion. Data were reported using descriptive statistics and bivariate analysis. Primary endpoint: rCDI within 2-8 weeks post-OVP completion. Secondary endpoints: vancomycin-resistant *Enterococcus *spp (VRE) in clinical culture post-OVP and 1-year mortality.

**Results:**

70 patients included: 32 (46%) no-OVP and 38 (54%) OVP. Baseline characteristics, previous CDI treatment, and outcomes were similar (Table 1). Index CDI was severe in the OVP group (18, 47% vs. 9, 28%). Median Charlson comorbidity index: 7 (3 – 9) no-OVP and 7 (5 – 9) OVP. OVP regimens, 125 mg by mouth: once daily 4 (10%), twice daily 22 (58%), and every 6 hours 12 (32%). Median prophylaxis duration: 10 days (6 – 13). rCDI occurred in 3 (9%) no-OVP and 2 (5%) OVP (*P* = 0.654). Mortality: 10 (31%) no-OVP and 16 (42%) OVP (*P* = 0.458).

Table 1. Patient Characteristics and Endpoints

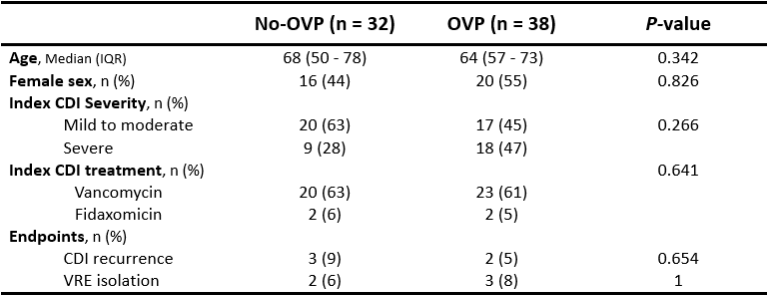

**Conclusion:**

OVP was utilized in approximately half of patients who required non-CDI antibiotics. Efficacy interpretation is limited by inconsistent dosing regimens and significant comorbid illness in the cohort. Future work will focus on further optimizing the BPA and standardizing the OVP regimen.

**Disclosures:**

**Rachel Kenney, PharmD**, **Medtronic, Inc.** (Other Financial or Material Support, spouse is an employee and shareholder) **Susan L. Davis, PharmD**, Nothing to disclose

